# Reversible Crosslinking of Polymer/Metal-Ion Complexes for a Microfluidic Switch

**DOI:** 10.1021/acsomega.1c04055

**Published:** 2021-12-14

**Authors:** Hojun Lee, Soon-Bo Kang, Hyunjae Yoo, Hae-Ryung Lee, Jeong-Yun Sun

**Affiliations:** † Department of Materials Science and Engineering, Seoul National University, 1 Gwanak-ro, Gwanak-gu, 151-744 Seoul, Republic of Korea; ‡ Research Institute of Advanced Materials (RIAM), Seoul National University, 1 Gwanak-ro, Gwanak-gu, 151-742 Seoul, Republic of Korea

## Abstract

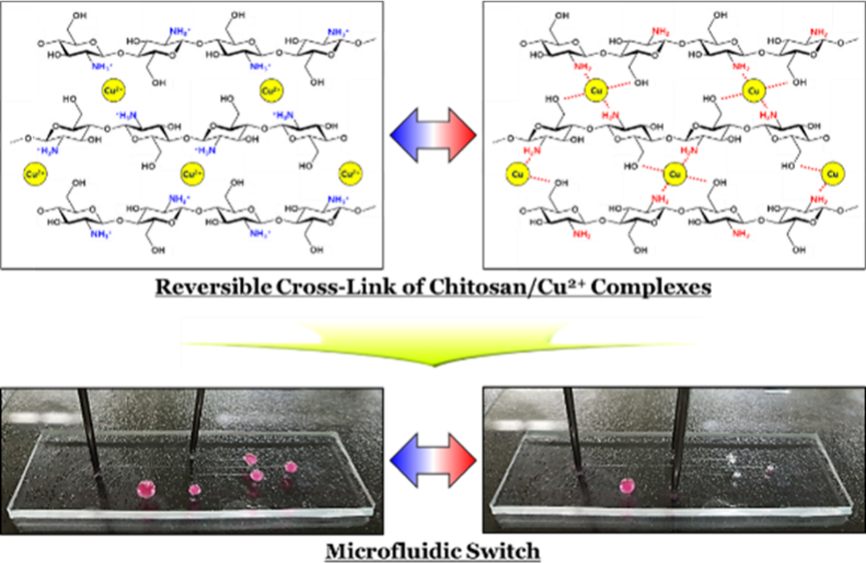

The importance of chitosan has been strongly emphasized in literature because this natural polymer could not only remove heavy metal ions in water but also have the potential for recyclability. However, reversible phase transition and its dynamics, which are highlighting areas of a recycle process, have not been studied sufficiently. Here, we present dynamic studies of the dissolution as well as the gelation of a physically crosslinked chitosan hydrogel. Specifically, a one-dimensional gel growth system and an acetate buffer solution were prepared for the precise analysis of the dominant factors affecting a phase transition. The dissolution rate was found to be regulated by three major factors of the pH level, Cu^2+^, and NO_2_^–^, while the gelation rate was strongly governed by the concentration of OH^–^. Apart from the gelation rate, the use of Cu^2+^ led to the rapid realization of gel characteristics. The results here provide strategies for process engineering, ultimately to determine the phase-transition rates. In addition, a microfluidic switch was successfully operated based on a better understanding of the reversible crosslinking of the chitosan hydrogel. Rapid gelation was required to close the channel, and a quick switchover was achieved by a dissolution enhancement strategy. As a result, factors that regulated the rates of gelation or dissolution were found to be useful to operate the fluidic switch.

## 1 Introduction

Chitosan is a polysaccharide obtained from the deacetylation of chitin.^1−3^ Based on its unique characteristics, such as hydrophilicity, biocompatibility, and pH responsivity, a wide range of applications, such as drug delivery and sensors, have been developed.^4−9^ Especially, this polymer is also capable of capturing heavy metal ions through a coordination bond, so toxic metal ions in aqueous solutions can be easily removed by a chitosan/metal-ion complex.^10,11^ Furthermore, a recyclable water purification system could be achieved using a reversible crosslinking of the chitosan hydrogel. This indicates that chitosan is certainly attractive because this natural polymer could reduce the environmental concern as well.

**Figure 1:**
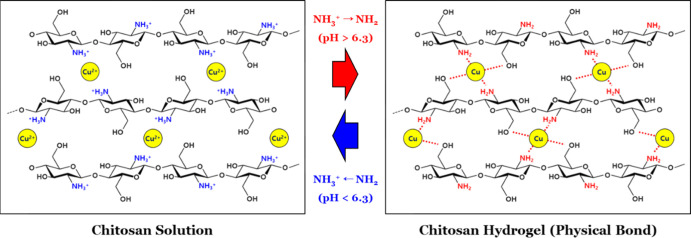
Illustration of the reversible crosslinking of the physically crosslinked chitosan/Cu^2+^ hydrogel.

In relation to chitosan, recyclability could be achieved by a reversible phase transition of physically crosslinked polymers. For example, chitosan becomes dissolved when it is exposed to an acid.^12−16^ This is mainly due to water solubility changes determined by the pH level. In detail, chitosan polymers become soluble, accompanying the protonation of NH_2_ below pH 6.3 and start to form a gel due to the deprotonation of NH_3_^+^ above pH 6.3.^13−17^ Similarly, coordination bonds between chitosan and transition-metal ions are also related to the pH levels of the solutions. For example, the chitosan/Cu^2+^ hydrogel was obtained above pH 6.3, while the chitosan polymer was soluble in aqueous solutions at the pH level below 6.3 (Figure 1).^18^ This is explained by lone-pair electrons which are donated from NH_2_ to transition-metal ions to facilitate their temporary sharing. Therefore, a fundamental task is to determine the behavior of the polymer at different pH levels.

With regard to the gelation of the chitosan hydrogel, previous research studies were focused on the gelation rate using a one-dimensional gel growth system in an alkali solution.^3,19−21^ Nie et al. reported that a high concentration of NaOH in an alkali led to an elevated growth rate of the chitosan/Cu^2+^ hydrogel. Interestingly, the rapid growth led to an oriented fibrous structure, while a discrete multi-layer was obtained at a low growth rate.^19^ Comparably, Dobashi et al. discussed the gelation speed of chitosan polymers in alkalis with theoretical phase-transition dynamics approaches.^20^ However, more in-depth studies are still required to clarify the factors as well as the mechanism of chitosan gelation. Fortunately, the color shift of the chitosan/metal-ion complexes is supposed to be a great hint to explain the gelation process.

On the other hand, a few research groups reported chitosan derivatives obtained by chemical modifications for the sake of enhancing the solubility of the chitosan powder.^22−25^ For example, Kim et al. suggested that a catechol conjugation process could enhance the solubility of modified chitosan powder in an aqueous solution at pH values up to 7.0.^22^ However, previous works did not discuss the case from a hydrogel to a solution as the concept of a reversible phase transition. Besides, the effect of metal-ion binding to the chitosan polymer on the dissolution has been barely studied compared to the gelation. We believe that a comprehensive understanding of the dissolution behavior will contribute to not only establishing the recycle process but also expanding the application field of the chitosan polymer.

Here, we have conducted dynamic studies of the dissolution as well as the gelation of physically crosslinked chitosan/metal-ion complexes. Among metal ions, Cu^2+^ was first considered due to the strong affinity of NH_2_ to Cu^2+^.^26,27^ It is noteworthy that the hydrogels obtained from the gelation test were directly applied to the dissolution test in order to assume the recycling process. Furthermore, a one-dimensional gel growth system and an acetate buffer solution were prepared for the precise analysis of the dominant factors to determine the rates of a phase transition. Lastly, a brief ligand chemistry was utilized in order to understand the interaction between the polymer and metal ions when a phase transition occurred. The effect of other metal ions (Mn^2+^, Fe^3+^, and Ca^2+^) was also investigated compared to Cu^2+^. At the end of the study, a microfluidic switch was operated based on the reversible crosslinking of the chitosan/Cu^2+^ hydrogel.

## 2 Results and Discussion

### 2.1 Gelation of the Chitosan/Cu^2+^ Hydrogel

**Figure 2:**
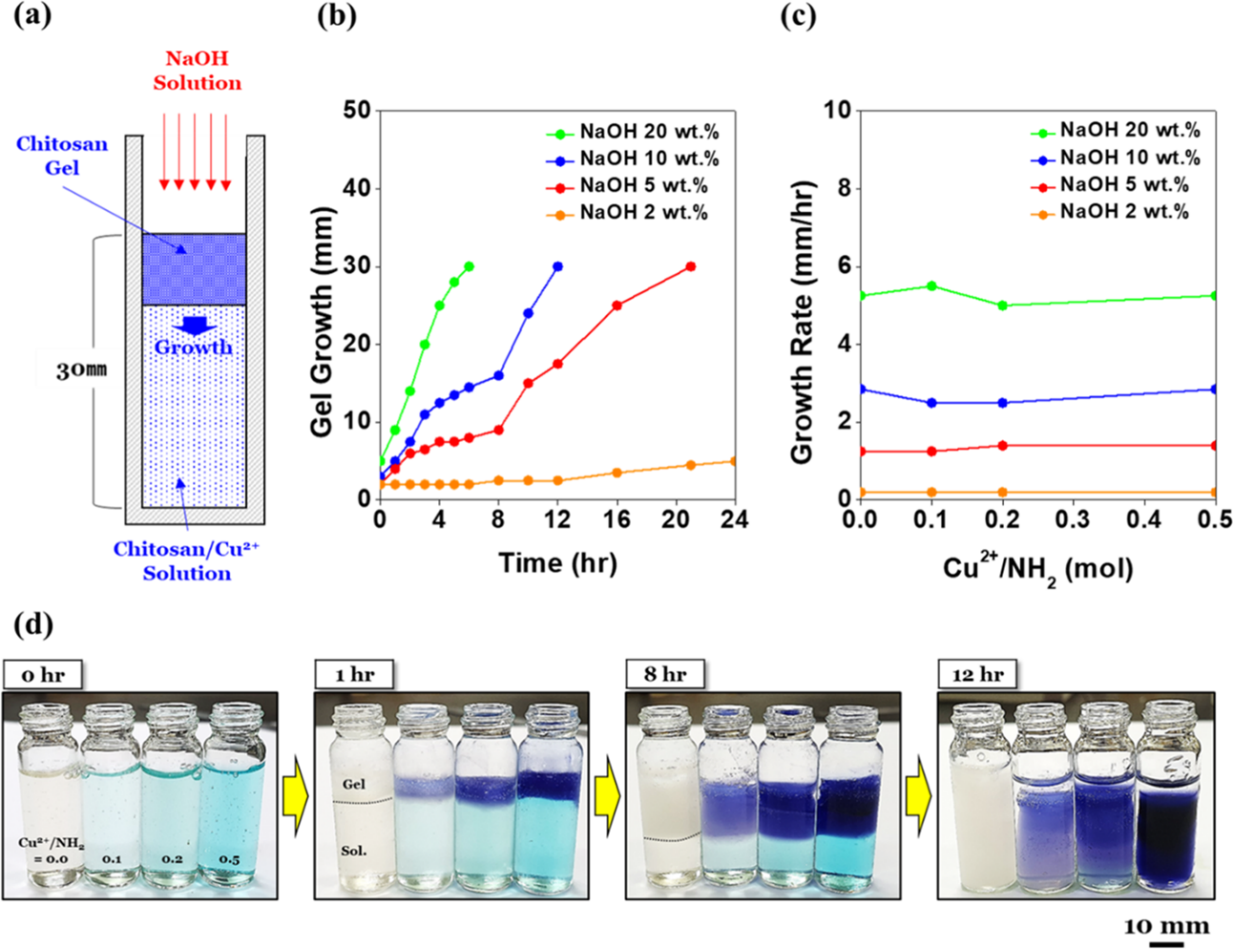
Gelation test of the chitosan/Cu^2+^ hydrogel: (a) schematic description of a one-dimensional chitosan/Cu^2+^ hydrogel grown in contact with the NaOH aqueous solution. (b) Time courses of the chitosan/Cu^2+^ hydrogel (Cu^2+^/NH_2_ = 0.2) with concentrations of 2–20 wt % NaOH over a time of 24 h. (c) Growth rate of chitosan/Cu^2+^ hydrogels (Cu^2+^/NH_2_ = 0.0–0.5) with concentrations of 2–20 wt % NaOH. (d) Photos of growing chitosan/Cu^2+^ hydrogel samples (Cu^2+^/NH_2_ = 0.0–0.5) in contact with a 10 wt % NaOH aqueous solution. NH_2_ caused higher energy absorption compared to H_2_O such that the color change from light blue (chitosan/Cu^2+^ solution) to deep blue (chitosan/Cu^2+^ hydrogel) occurred.

As shown in Figure 2a, the chitosan/Cu^2+^ hydrogel formation rate was analyzed through a one-dimensional gelation system. A glass mold filled with the chitosan/Cu^2+^ solution and a clotting bath in which NaOH was dissolved were both prepared. The chitosan/Cu^2+^ solution started to form a gel as soon as the glass mold was immersed in the clotting bath. As described in Figure 2d, a solution region under a gel layer decreased during gel growth and the completely grown chitosan/Cu^2+^ hydrogel was finally obtained. Then, a one-dimensional grown part was verified using the rheological characterization. The values of the storage modulus were always higher than those of the loss modulus regardless of Cu^2+^, which indicated that the hydrogel was successfully fabricated (Figure S1). Besides, the gelation process was visual because Cu^2+^ dissolved in the chitosan polymer solution took part in the gelation through coordination bonding between NH_2_ and Cu^2+^ considering the color change from light blue (chitosan/Cu^2+^ solution) to deep blue (chitosan/Cu^2+^ hydrogel). The origin of different colors is that NH_2_ caused more splitting of the d-orbital of Cu^2+^ than water (H_2_O), as the N atoms in NH_2_ were more electropositive than the O atoms in H_2_O. As a result, the chitosan/Cu^2+^ hydrogel absorbed higher energy corresponding to yellow light, the complementary color of deep blue.^28,29^

For the chitosan polymers, they are able to become a physically crosslinked gel when NH_3_^+^ is deprotonated to NH_2_ on aqueous surfaces under alkaline conditions. Therefore, the rate of deprotonation from NH_3_^+^ to NH_2_ should be a major factor affecting the chitosan hydrogel formation outcome. As shown in Figure 2b, the time courses of gel growth indicated that an increase in the concentration of NaOH led to rapid growth of the gel. This can be explained by the higher diffusion rate of OH^–^ through the grown hydrogel layer in order to deprotonate NH_3_^+^ at the sol/gel interface. The 2 wt % NaOH aqueous solution required more than 72 h until gelation was fully complete owing to the low diffusion rate of OH^–^. Apparently, the chitosan gel growth rate strongly depended on the concentration of NaOH. From a similar point of view, we predicted that a small amount of acetic acid in chitosan solution could also be supportive in order to promote the gel growth. According to the previous work reported by Dobashi et al., a large amount of acetic acid in the chitosan solution interrupted the chitosan gel growth due to a delay in the deprotonation of NH_3_^+^ at the sol/gel interface.^20^

On the other hand, the influence of the concentration of Cu^2+^ (Cu^2+^/NH_2_ = 0.0–0.5) on the gel formation rate with the same concentration of NaOH was mostly negligible (Figure 2c,d). This result was quite reasonable in that coordination bonding can form only when lone-pair electrons are shared between NH_2_ and Cu^2+^. Figure S2 clearly indicates that a chitosan/Cu^2+^ hydrogel with a large amount of Cu^2+^ (Cu^2+^/NH_2_ = 1.0) also grew at a speed identical to that of a pure chitosan hydrogel in the 20 wt % NaOH aqueous solution. Moreover, we investigated the gelation behavior with different metal ions (Cu^2+^, Mn^2+^, Fe^3+^, and Ca^2+^) incorporated with the chitosan polymer (Figure S3). In brief, the chitosan/metal-ion gel (metal ion/NH_2_ = 0.2) growth rate was undoubtedly accelerated when the concentration of NaOH became high. For example, the chitosan/Fe^3+^ hydrogel grew faster in 10 wt % NaOH aqueous solution compared to 5 wt % NaOH aqueous solution. However, each chitosan/metal-ion hydrogel grew at a different rate, which followed the order of Fe^3+^ ≈ Cu^2+^ > Mn^2+^ ≈ Ca^2+^. This was probably caused by the change of affinity of NH_2_ to metal ions. According the previous report, ethylenediamine favored Fe^3+^ and Cu^2+^ rather than Mn^2+^ and Ca^2+^.^27^ This probably resulted in byproducts such as Mn(OH)_2_ and Ca(OH)_2_, which interrupted the diffusion of OH^–^. As explained by Nie et al., Ca(OH)_2_ precipitation within the chitosan hydrogel was clearly observed.^19^ The result indicated that the strong affinity of NH_2_ to Cu^2+^ encouraged the growth of the chitosan/Cu^2+^ hydrogel.

**Figure 3:**
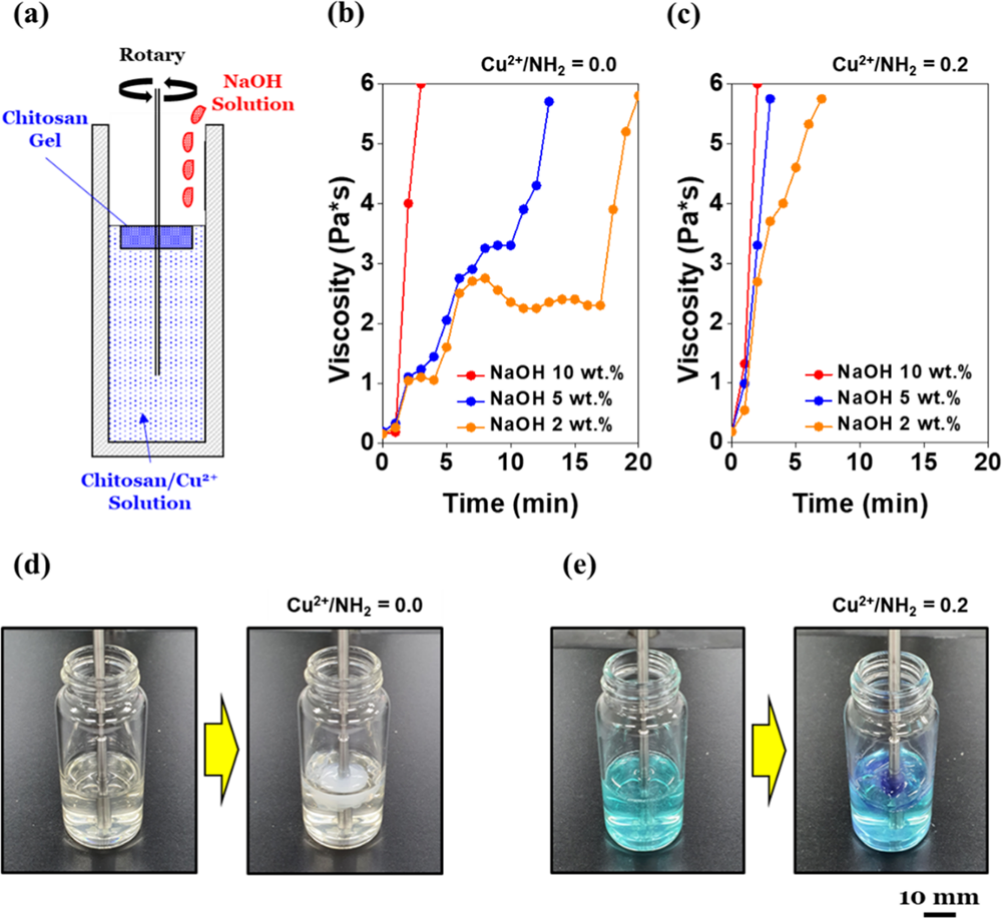
Viscosity measurements of the chitosan/Cu^2+^ hydrogel: (a) schematic description of a rotary viscometer system using the chitosan/Cu^2+^ hydrogel with sequential drops (0.3 mL/min) of a NaOH aqueous solution. (b) Time courses of the pure chitosan hydrogel (Cu^2+^/NH_2_ = 0.0) at concentrations of 0–10 wt % NaOH. (c) Time courses of the chitosan/Cu^2+^ hydrogel (Cu^2+^/NH_2_ = 0.2) at concentrations of 0–10 wt % NaOH. (d) Photos of the initial and final states of the pure chitosan hydrogel (Cu^2+^/NH_2_ = 0.0). (e) Photos of the initial and final states of the chitosan/Cu^2+^ hydrogel (Cu^2+^/NH_2_ = 0.2).

Next, the viscosity changes of the chitosan/Cu^2+^ complexes (Cu^2+^/NH_2_ = 0.0, 0.2) with sequential drops of aqueous NaOH solutions (0–10 wt %) were measured (Figure 3). As shown in Figure 3a, a rotary viscometer was utilized and the viscosity was recorded until the spindle became unrotated (Figure 3d,e). Even though the viscosity measurement was inadequate in spatially heterogeneous complexes, it could express a torque in the hydrogel, hindering the rotation of the spindle. In detail, a piece of hydrogel nucleated at the surface of the solution started to merge with each other and interrupt the rotation of the spindle so that the rapid gel formation led to a sharp increase in torque which was expressed by the viscosity. Therefore, the viscosity fluctuations of the chitosan/Cu^2+^ complex may indicate how rapidly the gel growth proceeded. However, we used representative viscosity measured in the whole sample because it was impossible to detect the values of the heterogeneous regions during gelation. Figure 3b,c clearly shows that the slope corresponding to the 10 wt % NaOH aqueous solution was much steeper than the others (2 and 5 wt %). This result indicates that gel characteristics were more quickly obtained when a higher concentration of NaOH aqueous solution was added. This can also be explained by the higher diffusion rate of OH^–^, resulting in rapid gel formation. It was quite analogous to the gelation test in Figure 2 in that the concentration of the NaOH aqueous solution determined the speed of the gelation. However, the time scale was shorter compared to Figure 2, because the viscosity of the complexes exceeded a maximum range of the measurement promptly during the gelation processes. Apart from the gel formation rate, it was also found that the chitosan/Cu^2+^ complex (Figure 3c) tended to show higher viscosity than the pure chitosan hydrogel (Figure 3b) when the same amount of NaOH aqueous solution was dropped. This was mainly due to the stronger crosslinking of chitosan polymers with the help of Cu^2+^, which greatly hinders the rotation of the spindle. The much higher storage modulus of the chitosan/Cu^2+^ hydrogel (Cu^2+^/NH_2_ = 0.2) than the pure chitosan hydrogel (Cu^2+^/NH_2_ = 0.0) could be other evidence of the strong crosslinking through coordination bonding (Figure S1).

In conclusion, the higher diffusion rate of OH^–^ was the dominant driving force of the physical gelation of chitosan/Cu^2+^ regardless of the concentration of Cu^2+^. However, the Cu^2+^ within the chitosan polymers accelerated the gaining of gel characteristics.

### 2.2 Dissolution of the Chitosan/Cu^2+^ Hydrogel

**Figure 4:**
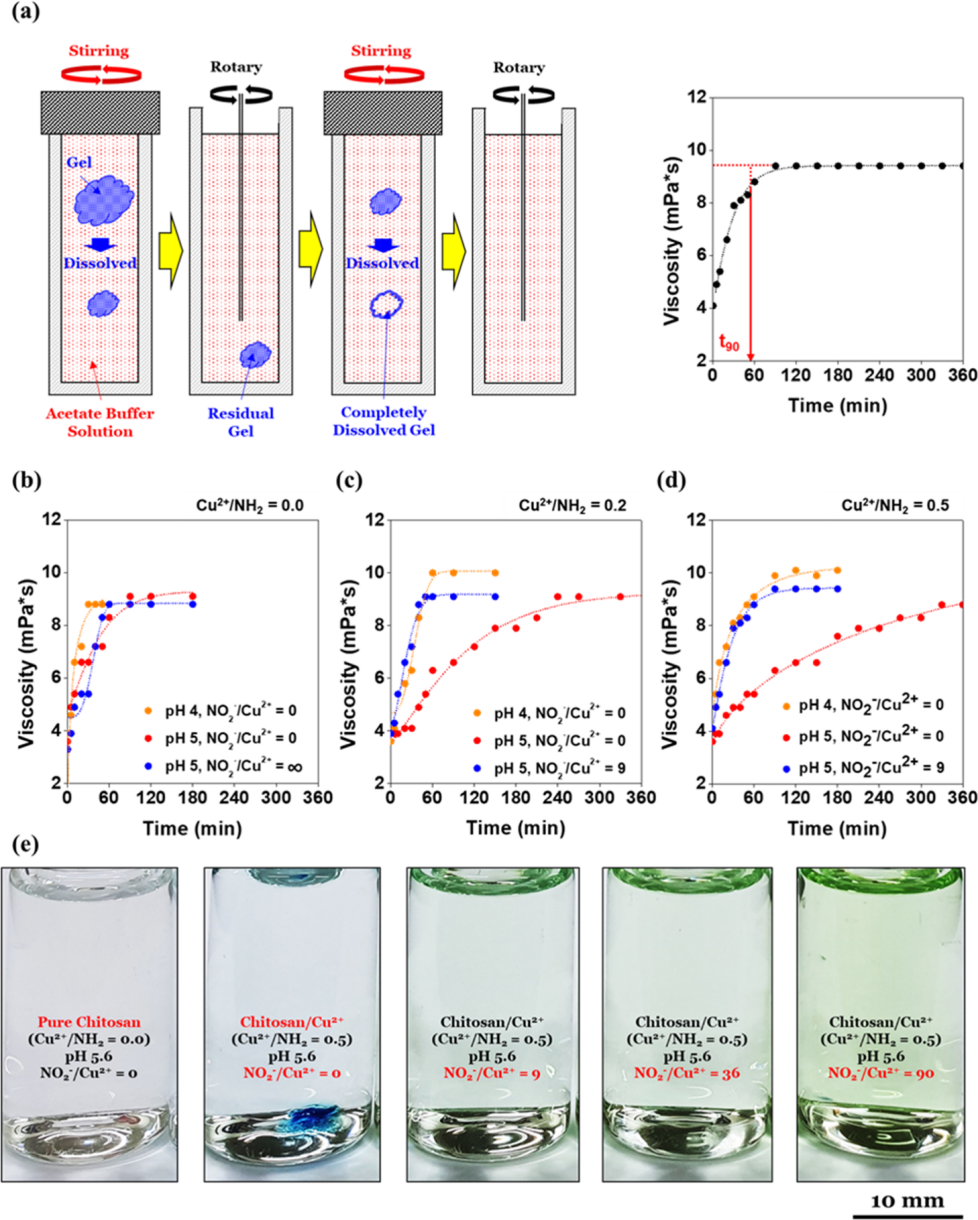
Viscosity measurements of the chitosan/Cu^2+^ hydrogel dissolution and solution color comparison: (a) schematic description of the chitosan/Cu^2+^ hydrogel dissolution using an acetate buffer solution system with pH levels ranging from 3.8 to 5.6 and viscosity measurements. Here, *t*_90_, the time to reach 90% of the converging value after Weibull fitting, was considered as the dissolution time. (b) Time courses of pure chitosan hydrogel (Cu^2+^/NH_2_ = 0.0) dissolution in acetate buffer solutions (pH 4 and 5 and NO_2_^–^/Cu = 0, ∞). (c) Time courses of chitosan/Cu^2+^ hydrogel (Cu^2+^/NH_2_ = 0.2) dissolution in acetate buffer solutions (pH 4 and 5 and NO_2_^–^/Cu = 0, 9). (d) Time courses of chitosan/Cu^2+^ hydrogel (Cu^2+^/NH_2_ = 0.5) dissolution in acetate buffer solutions (pH 4, 5 and NO_2_^–^/Cu = 0, 9). (e) Photos of solutions with different colors after chitosan/Cu^2+^ hydrogel (Cu^2+^/NH_2_ = 0.0, 0.5) dissolution in acetate buffer solutions (pH 5.6, NO_2_^–^/Cu^2+^ = 0–90). NO_2_^–^ caused higher energy absorption compared to NH_2_ such that the Cu/NO_2_^–^ aqueous solution was not bluish but greenish. The residual chitosan/Cu^2+^ hydrogel (Cu^2+^/NH_2_ = 0.5) was observed when NO_2_^–^ was not utilized at pH 5.6 (NO_2_^–^/Cu^2+^ = 0).

Before the dissolution test, chitosan/Cu^2+^ hydrogels with various compositions (Cu^2+^/NH_2_ = 0.0–0.5) were prepared using the 10 wt % NaOH aqueous solution with the one-dimensional gelation system. That is to say, the hydrogels obtained from the gelation test were applied to the dissolution test as the concept of the recyclable process. Then, factors that disintegrate a physically crosslinked chitosan/Cu^2+^ hydrogel were analyzed using 1.0 M acetate buffer solution system with pH levels from 3.8 to 5.6 accompanied by mechanical stirring (Figure 4a). This pH range should be suitable for a dissolution test of the chitosan hydrogel considering its p*K*_a_ value of 6.3. Even though citrate buffer solution could also provide the useful pH levels from 3.0 to 6.2, the chitosan polymer was not dissolved well, probably due to the strong ionic strength of the solution (Table S1).^30^ Besides, the viscosity was measured using a rotary viscometer in order to analyze the dissolution quantitatively. In detail, 1.0 g of the chitosan hydrogel was immersed in 10 mL of the buffer solution whose initial viscosity was 3.5 ± 0.7 mPa s. After that, the viscosity of the solution in which the chitosan polymer disintegrated gradually increased until the hydrogel was completely dissolved. Then, the viscosity uniformly converged at 9.4 ± 1.0 mPa s because the final concentration of the chitosan polymer was equal. Therefore, the convergence of the viscosity without any residual chitosan/Cu^2+^ hydrogel indicated the complete dissolution. Here, *t*_90_ was considered as the dissolution time. As shown in Figure 4a, *t*_90_ was defined as the time to reach 90% of the converging value after Weibull fitting. Even though the heterogeneous regions should exist during dissolution, we reported the viscosity measured in the solution representatively.

**Figure 5:**
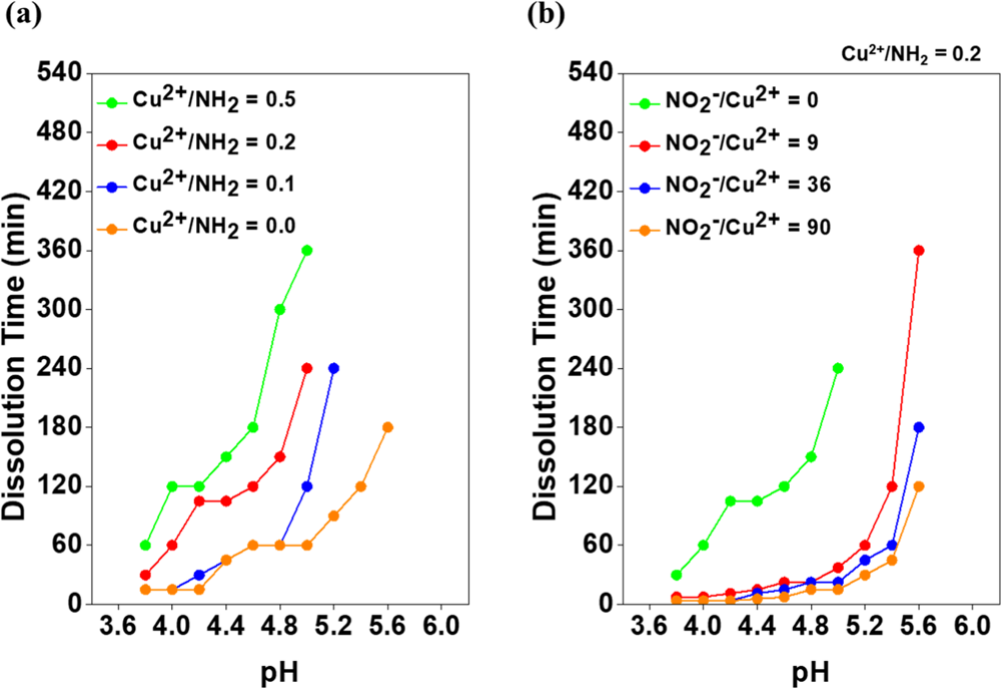
Dissolution test of the chitosan/Cu^2+^ hydrogel: (a) influence of Cu^2+^ within chitosan polymers on the chitosan/Cu^2+^ hydrogel (Cu^2+^/NH_2_ = 0.0–0.5) dissolution process. In this experiment, NO_2_^–^ was not included in the acetate buffer solution. (b) Influence of NO_2_^–^ which was dissolved in an acetate buffer solution (NO_2_^–^/Cu^2+^ = 0–90) on the chitosan/Cu^2+^ hydrogel (Cu^2+^/NH_2_ = 0.2) dissolution process.

Figure 5 shows the result of the dissolution test described in Figure 4. Contrary to gelation, the rate of protonation from NH_2_ to NH_3_^+^ should be a major factor affecting the chitosan hydrogel dissolution outcome. As shown in Figure 5a, a decrease in the pH level powered the dissolution rate significantly. Fortunately, there was no distinct change in the pH level during the dissolution of the chitosan/Cu^2+^ hydrogel due to the acetate buffer solution.

Next, two additional key factors which regulate the dissolution rate of the chitosan/Cu^2+^ hydrogel were discovered. The chitosan/Cu^2+^ hydrogel dissolved slowly as the concentration of Cu^2+^ increased. Moreover, Cu^2+^ led to a lower maximum pH level, at which point the chitosan/Cu^2+^ hydrogel was completely disintegrated. For example, the chitosan/Cu^2+^ hydrogel (Cu^2+^/NH_2_ = 0.2, 0.5) remained swollen at pH levels ranging from 5.2 to 5.6, where the pure chitosan hydrogel could be perfectly soluble (Figure 5a). This implies that strong binding within the polymers induced by coordination complexes required an intense driving force in order to disintegrate. Fortunately, the metal-ion affinity of molecules could be utilized for the purpose of enhancing the dissolution rate as a ligand exchange from weaker molecules to stronger molecules generally occurs in an aqueous solution.^31−33^ Given the primary requirements such as metal-ion affinity, water solubility, and p*K*_a_, NO_2_^–^ was regarded as an excellent molecule. Theoretically, N atoms in NO_2_^–^, which have electronegative O atoms, show stronger metal-ion affinity than N atoms in NH_2_ such that Cu^2+^ prefers NO_2_^–^ to NH_2_.^29,31−34^ Despite the fact that there are a few ligand molecules with stronger metal-ion affinity than NH_2_, they were not suitable for chitosan/Cu^2+^ hydrogel dissolution (Table S2). For example, 2,2′-bipyridine is insoluble in water and cyanide ions are weakly acidic with a p*K*_a_ of 9.2. Here, NaNO_2_ was used as the source of NO_2_^–^, and its high solubility in water was readily confirmed, more than 50 g/100 mL. As expected, NO_2_^–^ dissociated from NaNO_2_ enhanced the dissolution of the chitosan/Cu^2+^ hydrogel, and it is shown in Figure 5b. In detail, the dissolution rate increased dramatically as higher concentrations of NO_2_^–^ (NO_2_^–^/Cu^2+^ = 0–90) were utilized at the same pH level. In addition, the maximum pH level at which a fully disintegrated solution was obtained tended to be elevated when NO_2_^–^ was applied. For example, the chitosan/Cu^2+^ hydrogel (Cu^2+^/NH_2_ = 0.2) could be completely dissolved at pH levels ranging from 5.0 to 5.6 when NO_2_^–^ was utilized (NO_2_^–^/Cu^2+^ = 9–90), while it remained swollen at a pH level above 5.0 without NO_2_^–^ (NO_2_^–^/Cu^2+^ = 0). The strong effect of NO_2_^–^ was investigated when other metal ions (Mn^2+^, Fe^3+^, and Ca^2+^) were utilized instead of Cu^2+^ (Table S3). For example, it took at most 50 min to dissolve the chitosan/Fe^3+^ hydrogel (Fe^3+^/NH_2_ = 0.2) at pH 5 with NO_2_^–^ (NO_2_^–^/Mn^2+^ = 90), while the same hydrogel was completely disintegrated after 450 min at pH 5 without NO_2_^–^. However, it was especially notable that metal ions apparently affected the dissolution rate of the chitosan/metal-ion hydrogels, probably due to the different affinity of NH_2_ to metal ions.^26,27^ For example, the chitosan/Ca^2+^ hydrogel tended to be dissolved faster than others, and this was probably because of the weak affinity of NH_2_ to Ca^2+^ caused by the absence of the d-orbital (not the transition metal).^19^

Interestingly, NO_2_^–^ did not work when the pure chitosan hydrogel was dissolved (Cu^2+^/NH_2_ = 0.0). For example, a converging time at pH 5 was not significantly affected by NO_2_^–^ (NO_2_^–^/Cu^2+^ = 0, ∞), as shown in Figure 4b. This was a major difference in that NO_2_^–^ (NO_2_^–^/Cu^2+^ = 9) shortens the converging time of the viscosity when chitosan/Cu^2+^ (Cu^2+^/NH_2_ = 0.2, 0.5) was dissolved (Figure 4c,d). Figure S4 shows the significant effect of NO_2_^–^ on the chitosan/Cu^2+^ hydrogel dissolution visually. This can be explained by the interaction between the ligand molecules and the metal ions. In detail, Cu^2+^ could be favorably extracted from strong binding to chitosan polymers with the aid of NO_2_^–^, which has stronger metal-ion affinity compared to NH_2_,^29,31−34^ while the pure chitosan polymer was not affected by the ligand molecules. In fact, different colors of the chitosan/Cu^2+^ solution which considerably depended on the ligand molecules could be other evidence of the suggested mechanism. As shown in Figure 4e, chitosan/Cu^2+^ dissolved in the CH_3_COOH/NO_2_^–^ aqueous solution was light green in color,^35−37^ while chitosan/Cu^2+^ dissolved in the CH_3_COOH aqueous solution was light blue. The reason for the different colors can be explained as in Section 2.1. In practice, the color of the Cu^2+^/NO_2_^–^ (CuCl_2_/NaNO_2_) aqueous solution was greenish, whereas the Cu^2+^/NH_2_ (ethylenediamine) aqueous solution was bluish. However, the dissolution of the chitosan/Cu^2+^ hydrogel was not expected at a pH level above 6.3, even with NO_2_^–^, mainly because chitosan polymers could not become hydrated unless protonation occurred.

In conclusion, two other factors can clearly be involved when the chitosan/Cu^2+^ hydrogel was intended to be dissolved at a pH below 5.6. Cu^2+^, which contributed to stronger crosslinking, led to a slower dissolution, while NO_2_^–^, which tended to attract Cu^2+^, accelerated the dissolution process dramatically. Particularly, NO_2_^–^ could help the chitosan/Cu^2+^ hydrogel dissolve faster compared to the pure chitosan hydrogel.

### 2.3 Reversible Crosslinking for a Fluidic Switch

A fluidic switch for water flow regulation was operated by means of the reversible crosslinking of the chitosan/Cu^2+^ hydrogel. Figure S5 shows the simple construction of the fluidic switch system which was operated via the reversible crosslinking of chitosan/Cu^2+^ complexes.

**Figure 6:**
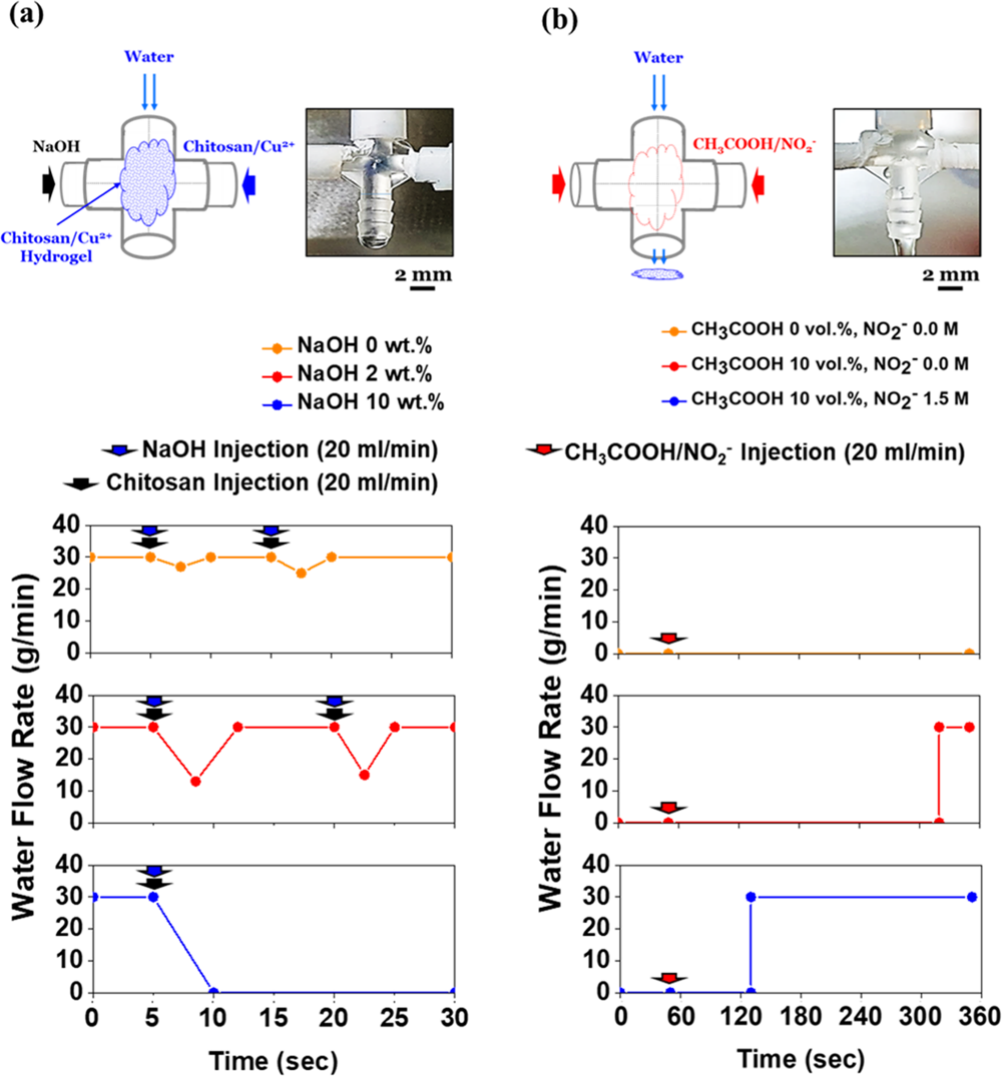
Fluidic switch test with reversible crosslinking of the chitosan/Cu^2+^ hydrogel: the water flow rate was fixed at 30 mL/min. (a) Close test (gelation) with concentrations of 0–10 wt % NaOH. The chitosan/Cu^2+^ solution and a NaOH aqueous solution were injected simultaneously to form a gel. (b) Open test (dissolution) with a CH_3_COOH (0, 10 wt %)/NO_2_^–^ (0, 1.5 M) aqueous solution after the channel in (a) was blocked.

A water flow at a rate of 30 mL/min was blocked only when gelation occurred at the connector intersection. Then, all the water (30 mL/min) started to flow along the detour route in 5 s (Figure 6a, blue symbol). In order to form the gel, the 0.02 g/mL chitosan/Cu^2+^ solution (Cu^2+^/NH_2_ = 0.2) and a 10 wt % NaOH aqueous solution were injected simultaneously at a rate of 20 mL/min from opposite directions perpendicular to the water flow line. A blue gel then successfully formed despite the continual supply of water (Figure 6a). However, the fluidic switch did not work when an insufficient amount of OH^–^ was injected due to the slow gel formation rate. For example, the 2 wt % NaOH aqueous solution did not result in rapid gel formation such that water continued to flow with a slight hindering of the flow rate (Figure 6a, red symbol).

After the water flow was blocked by successful gel formation, an open test was implemented. The water flow rate increased instantly to 30 mL/min as soon as the gel was removed when the CH_3_COOH/NO_2_^–^ aqueous solution was steadily injected instead of the chitosan/Cu^2+^ solution and the NaOH aqueous solution. In addition, partially dissolved chitosan polymers were readily found at the outlet. Interestingly, a high concentration of CH_3_COOH and NO_2_^–^ tended to shorten the operating time until the channel opened completely, while pure water at a rate of 30 mL/min from the water supply was not capable of removing the chitosan/Cu^2+^ hydrogel, which blocked the water flow (Figure 6b). This indicates that the chitosan/Cu^2+^ hydrogel not only was strong enough to endure the solvent pressure but was also reversible for a fluidic switch application. However, a small area of the aqueous surface in acidic conditions and the absence of mechanical stirring led to a slow switchover from closed to open. For example, it took more than 70 s until the channel was fully opened even when 1.5 M of NO_2_^–^ was utilized (Figure 6b, blue symbol). Note that Y-shaped connectors were used when injecting the CH_3_COOH/NO_2_^–^ aqueous solution in order to verify the effect of the dissolution rate clearly without pressure on the blocking gel.

As a result, the fluidic switch was demonstrated based on the reversible phase transition of the chitosan/Cu^2+^ hydrogel. Furthermore, factors that regulated the rates of gelation or dissolution were found to be useful when operating the fluidic switch.

### 2.4 Microfluidic Switch

**Figure 7:**
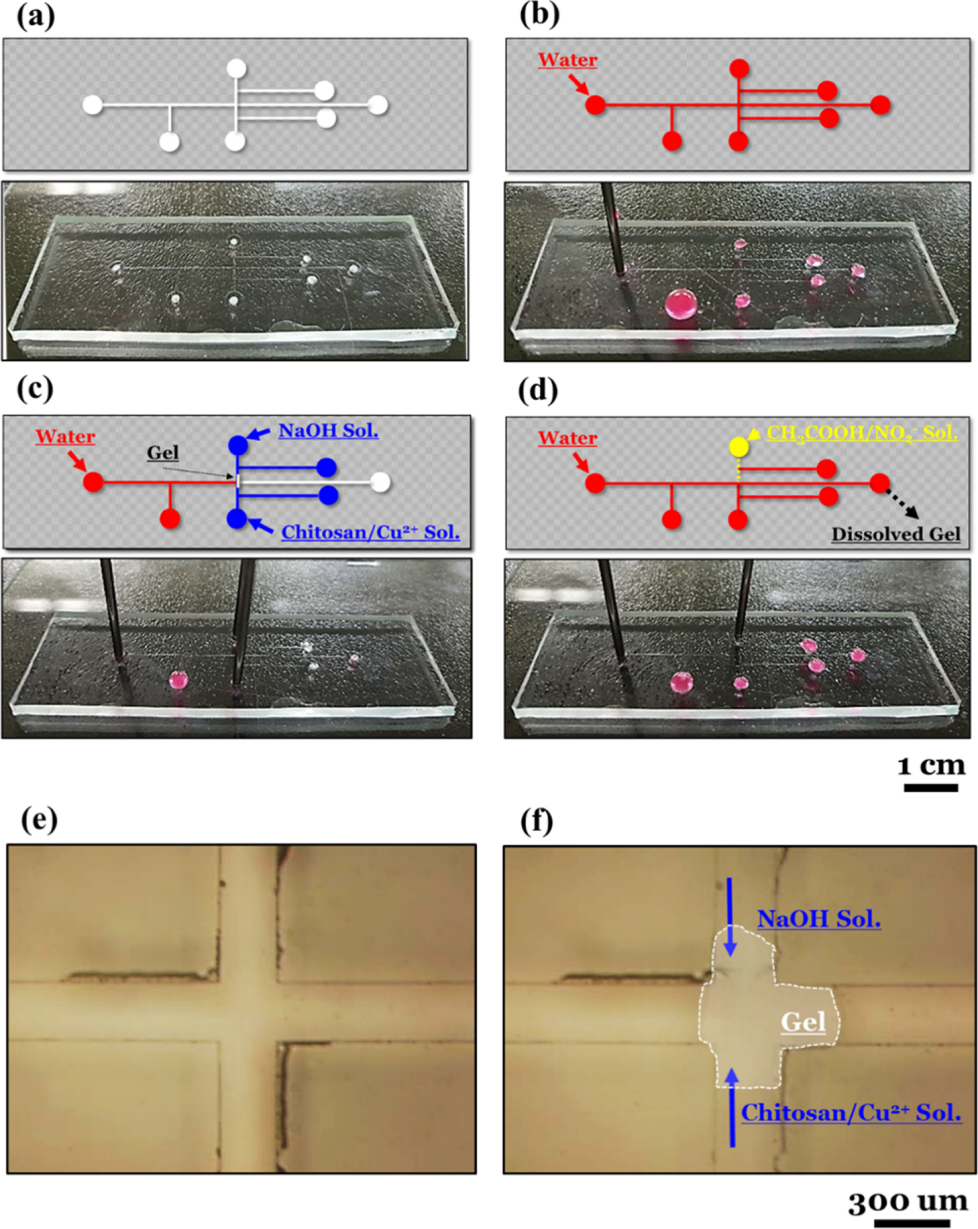
Microfluidic switch test: (a) seven-hole microfluidic channel using patterned PDMS on glass. The channel dimensions were fixed as 300 μm (width) and 50 μm (depth). (b) Initial water flow check with a rate of 150 μL/min before the switch test. (c) The channel was closed by the gelation of chitosan/Cu^2+^ near the intersection. All the water started to flow along the detour route in 5 s. (d) The channel opened in 15 s due to the dissolution of the chitosan/Cu^2+^ hydrogel using the CH_3_COOH/NO_2_^–^ aqueous solution. (e) Microscopy image of the intersection before the switch test. (f) Microscopy image of the intersection after closing. The hydrogel is indicated by the white box to describe it more clearly.

For a space-saving device to be used in narrow and sophisticated channels, the solution-based fluidic switch system is expected to be advantageous compared to a mechanical valve or a tiny gear.^38−43^ As a demonstration, a seven-hole microfluidic channel was fabricated using patterned PDMS on a glass substrate (Figure 7a,7e). The channel structure was designed using a facile photolithography technique, and the attachment of PDMS/glass was achieved after a plasma treatment (Figure S6).^44−47^ The final dimensions of the channel were set to 300 μm × 50 μm (width × depth), and an initial water flow check was implemented before the switch test (Figure 7b).

When the 0.005 g/mL chitosan/Cu^2+^ solution and the 10 wt % NaOH aqueous solution were injected from both sides, all the water (150 μL/min) started to flow along the detour route in 5 s (Figure 7c). Figure 7f shows the chitosan/Cu^2+^ hydrogel part which directly formed near the intersection immediately after the injection of the two solutions. In particular, the gel was not removed until the acidic aqueous solution was injected instead of the NaOH aqueous solution. For example, the CH_3_COOH/NO_2_^–^ aqueous solution with a final concentration of 10 wt %/1.5 M led to a smooth water flow without any congestion in 15 s (Figure 7d). One aspect that differed from the fluidic switch described in Section 2.3 was the quick switchover from closed to open. We considered that this arose due to the low concentration of the chitosan/Cu^2+^ solution. In fact, a chitosan solution with high viscosity could not be injected through the microfluidic channel. Therefore, we held its concentration in the range from 0.02 to 0.005 g/mL. From a simple estimation, there was approximately 25% of NH_2_ to be protonated compared to the case described in Section 2.3.

As a result, this demonstration indicates that the reversible crosslinking of the chitosan/Cu^2+^ hydrogel can be applied to a microfluidic switch to regulate a water flow readily. Furthermore, reproducibility as well as quick switchover can be achieved in a microfluidic channel.

## 3 Conclusions

In summary, dominant factors which influence the rates of the reversible crosslinking of chitosan/Cu^2+^ hydrogels were studied experimentally. Rapid gel formation could be achieved when the concentration of OH^–^ increased regardless of the concentration of Cu^2+^, mainly due to the higher diffusion rate of OH^–^, which deprotonated NH_3_^+^ to NH_2_. Apart from the gelation rate, Cu^2+^ helped to realize gel characteristics quickly. Contrary to gelation, a decrease in the pH level was required to power the dissolution rate with the accelerated protonation of NH_2_ to NH_3_^+^. Furthermore, NO_2_^–^, which has strong affinity to metal ions, helped to disintegrate the chitosan/Cu^2+^ hydrogel well. This enhanced the dissolution rate of the chitosan/Cu^2+^ hydrogel. Even though the affinity of NH_2_ to metal ions (Mn^2+^, Fe^3+^, and Ca^2+^) apparently affected the dissolution as well as the gelation, a phase transition of the chitosan/metal-ion hydrogel was similarly controlled by the factors such as NaOH, pH, and NO_2_^–^. Finally, a fluidic switch was operated via the reversible crosslinking of chitosan/Cu^2+^ hydrogels. The gel which formed at a high concentration of OH^–^ blocked the water flow perfectly. This gel could not be removed until an acidic aqueous solution was injected. Furthermore, NO_2_^–^ dissolved in an acid aqueous solution shortened the operating time from closed to open. Moreover, a switch system could be successfully applied to a microfluidic channel as well. We expect that a controllable microfluidic device could be potentially applied to various fields such as hemostatic systems and trace metal analysis. Besides, this study provides a better understanding of the reversible crosslinking of the chitosan/metal-ion hydrogel and helps with the preparation of comprehensive strategies for the recycle process.

## 4 Experimental Section

### 4.1 Materials

Chitosan (Sigma, 448877) was used as a raw hydrogel material. The average viscosity of chitosan is 500 cp (1 wt % in 1 vol % CH_3_COOH aqueous solution), and the degree of deacetylation is 75–85%. Acetic acid (CH_3_COOH), sodium acetate (CH_3_COONa), sodium hydroxide (NaOH), copper chloride (CuCl_2_), manganese chloride (MnCl_2_), iron chloride (FeCl_3_), calcium chloride (CaCl_2_), and sodium nitrite (NaNO_2_) were purchased from Daejung Chemical and Metals Co., Ltd. and were of analytical reagent grade.

### 4.2 Gelation Test

Chitosan solution was prepared by dissolving chitosan powder in 2 vol % of a CH_3_COOH aqueous solution, with the final concentration of chitosan fixed at 2 wt %. Then, various amounts of CuCl_2_ powder were added in order to form a chitosan/Cu^2+^ solution with different molar ratios between Cu^2+^ and NH_2_ (Cu^2+^/NH_2_ = 0.0–0.5). Each solution was added to a glass mold before being immersed in a clotting bath. Clotting baths were prepared by dissolving various amounts of NaOH pellets in deionized water, with the final concentrations held in the range of 2–20 wt %. The growth of the chitosan/Cu^2+^ hydrogel was started when the chitosan/Cu^2+^ solution was immersed into the NaOH clotting bath. Each of the glass molds (2.5 mL of chitosan/Cu^2+^ solution) should be immersed in at least 500 mL of the NaOH aqueous solution in order to ensure uniform gelation. The chitosan/Cu^2+^ hydrogel growth rate was calculated according to the gel thickness, which was measured every hour. The color change from light blue (chitosan/Cu^2+^ solution) to deep blue (chitosan/Cu^2+^ hydrogel) was observed when the gelation proceeded.

### 4.3 Viscosity Measurements

The NaOH aqueous solution (2–10 wt %) as well as the chitosan/Cu^2+^ solution (Cu^2+^/NH_2_ = 0.0, 0.2) were prepared using the method described in Section 4.2. 40 mL of the chitosan/Cu^2+^ solution was added to a glass mold, and a spindle which was connected to a rotary viscometer (LVDV-II+, Brookfield) was dipped into the solution. The viscosity of the complex was recorded by adding an aqueous solution of NaOH with a speed of 0.3 mL/min.

### 4.4 Dissolution Test

Chitosan/Cu^2+^ hydrogels were prepared using the method described in Section 4.2 except that the final concentration of NaOH was fixed at 10 wt %. After gelation, chitosan/Cu^2+^ hydrogels were rinsed with deionized water repeatedly and stabilized in a deionized water bath for 24 h before the dissolution test. 1.0 M acetate buffer solutions with pH levels ranging from 3.8 to 5.6 were prepared in order to clarify the effect of the pH on the dissolution speed of the chitosan/Cu^2+^ hydrogel. The relative concentrations of CH_3_COOH and CH_3_COONa at each pH level were calculated with the Henderson–Hasselbalch equation (pH = p*K*_a_ + log{[*A*^–^]/[HA]}, p*K*_a_ for acetic acid = 4.75). The prepared buffer solutions were checked with a pH meter (Star A2116, Thermo Scientific). Then, various amounts of NaNO_2_ powder were added with different molar ratios between NO_2_^–^ and Cu^2+^ (NO_2_^–^/Cu^2+^ = 0–90). The prepared chitosan/Cu^2+^ hydrogels were immersed in buffer solutions with a stirring speed of 300 rpm at room temperature. Every 1.0 g of the chitosan/Cu^2+^ hydrogel should be immersed in 10 mL of the buffer solution under each condition. In order to confirm the complete disintegration quantitatively, the viscosity of the environmental buffer solution was measured using a rotary viscometer. The viscosity gradually increased as the chitosan hydrogel disintegrated into the buffer solution. Finally, the complete dissolution was determined when the viscosity became constant without any residual chitosan/Cu^2+^ hydrogel. Here, *t*_90_, the time to reach 90% of the converging value after Weibull fitting, was considered as the dissolution time.

### 4.5 Fluidic Switching Test

A cross-shaped fluidic test tube was assembled with transparent polypropylene tubes and a four-way connector. Both tubes and the connector are capable of operating in aqueous solutions ranging from the acidic to basic pH level. The inner radius of the four-way connector was 1 mm. Water flowed through a vertical line at a rate of 30 mL/min. Other tubes perpendicular to the water flow line were connected to syringes with chitosan/Cu^2+^ solution and NaOH solution. To close the water line, 0.02 g/mL of the chitosan/Cu^2+^ solution (Cu^2+^/NH_2_ = 0.2) and NaOH (0, 2, 10 wt %) aqueous solutions were simultaneously injected. The injection rate for both syringes was fixed at 20 mL/min. The water flow rate was calculated according to the amount of water which flowed in an operating interval. To reopen the water line, a CH_3_COOH (0, 10 vol %)/NO_2_^–^ (0.0, 1.5 M) aqueous solution was used. A Y-shaped bypass was connected to make a static flow of CH_3_COOH/NO_2_^–^ solution near the blocking gel. 20 mL/min was used as an injection speed of CH_3_COOH/NO_2_^–^ solution.

### 4.6 Microfluidic Switching Test

A seven-hole microfluidic channel was fabricated by patterning PDMS (Sylgard 184, Dow Corning) on glass. The patterned PDMS was developed by a facile photolithography technique in order to control the channel structure. The final dimensions of the microfluidic channel were fixed at 300 μm × 50 μm (width × depth). Permanent bonding between PDMS and the glass was obtained via a plasma bonding step after rinsing these surfaces with an acetone/ethanol solution. Additional heat (60 °C) and pressure (6500 N/m^2^) were applied to the PDMS/glass device for 48 h. The microfluidic switching test was operated under similar conditions in Section 4.5, except for the final concentration of chitosan/Cu^2+^ solution (0.005 g/mL, Cu^2+^/NH_2_ = 0.2) and flow rate (150 μL/min).

## Supporting Information Available

Rheological properties, gelation test of chitosan/Cu^2+^ and chitosan/metal-ion hydrogels, aqueous buffer solutions, ligand molecules, dissolution test of chitosan/Cu^2+^ and chitosan/metal-ion hydrogels, fluidic switch test, and schematic description of microfluidic channel fabrication (PDF)

## References

[ref1] Fu J.; Yang F.; Guo Z. The chitosan hydrogels: From structure to function. New J. Chem. 2018, 42, 17162–17180. 10.1039/c8nj03482f.

[ref2] Gandini A. Polymers from renewable resources: A challenge for the future of macromolecular materials. Macromolecules 2008, 41, 9491–9504. 10.1021/ma801735u.

[ref3] Nie J.; Lu W.; Ma J.; Yang L.; Wang Z.; Qin A.; Hu Q. Orientation in multi-layer chitosan hydrogel: morphology, mechanism and design principle. Sci. Rep. 2015, 5, 7635 10.1038/srep07635. 25559867 PMC4284508

[ref4] Berger J.; Reist M.; Mayer J. M.; Felt O.; Gurny R. Structure and interactions in chitosan hydrogels formed by complexation or aggregation for biomedical applications. Eur. J. Pharm. Biopharm. 2004, 57, 35–52. 10.1016/s0939-6411(03)00160-7. 14729079

[ref5] Nisar S.; Pandit A. H.; Wang L.-F.; Rattan S. Strategy to design a smart photocleavable and pH sensitive chitosan based hydrogel through a novel crosslinker: A potential vehicle for controlled drug delivery. RSC Adv. 2020, 10, 14694 10.1039/C9RA10333C. PMC9052095 35497171

[ref6] Hu L.; Sun Y.; Wu Y. Advances in chitosan-based drug delivery vehicles. Nanoscale 2013, 5, 3103–3111. 10.1039/c3nr00338h. 23515527

[ref7] Sadat Ebrahimi M. M.; Schönherr H. Enzyme-sensing chitosan hydrogels. Langmuir 2014, 30, 7842–7850. 10.1021/la501482u. 24914451

[ref8] Jang J.; Kang K.; Raeis-Hosseini N.; Ismukhanova A.; Jeong H.; Jung C.; Kim B.; Lee J. Y.; Park I.; Rho J. Self-Powered Humidity Sensor Using Chitosan-Based Plasmonic Metal-Hydrogel-Metal Filters. Adv. Opt. Mater. 2020, 8, 1901932 10.1002/adom.201901932.

[ref9] Kim J.-N.; Lee J.; Lee H.; Oh I.-K. Stretchable and self-healable catechol-chitosan-diatom hydrogel for triboelectric generator and self-powered tremor sensor targeting at Parkinson disease. Nano Energy 2021, 82, 105705 10.1016/j.nanoen.2020.105705.

[ref10] Lone S.; Yoon D. H.; Lee H.; Cheong I. W. Gelatin-chitosan hydrogel particles for efficient removal of Hg(ii) from wastewater. Environ. Sci.: Water Res. Technol. 2019, 5, 83–90. 10.1039/c8ew00678d.

[ref11] Upadhyay U.; Sreedhar I.; Singh S. A.; Patel C. M.; Anitha K. L. Recent advances in heavy metal removal by chitosan based adsorbents. Carbohydr. Polym. 2021, 251, 117000 10.1016/j.carbpol.2020.117000. 33142569

[ref12] Xu H.; Matysiak S. Effect of pH on chitosan hydrogel polymer network structure. Chem. Commun. 2017, 53, 7373–7376. 10.1039/c7cc01826f. 28612070

[ref13] Liu H.; Wang C.; Zou S.; Wei Z.; Tong Z. Simple, reversible emulsion system switched by pH on the basis of chitosan without any hydrophobic modification. Langmuir 2012, 28, 11017–11024. 10.1021/la3021113. 22762435

[ref14] Wu L.-Q.; Gadre A. P.; Yi H.; Kastantin M. J.; Rubloff G. W.; Bentley W. E.; Payne G. F.; Ghodssi R. Voltage-dependent assembly of the polysaccharide chitosan onto an electrode surface. Langmuir 2002, 18, 8620–8625. 10.1021/la020381p.

[ref15] Hu P.; Raub C. B.; Choy J. S.; Luo X. Modulating the properties of flow-assembled chitosan membranes in microfluidics with glutaraldehyde crosslinking. J. Mater. Chem. B 2020, 8, 2519–2529. 10.1039/c9tb02527h. 32124900

[ref16] Deng Y.; Ren J.; Chen G.; Li G.; Wu X.; Wang G.; Gu G.; Li J. Injectable in situ cross-linking chitosan-hyaluronic acid based hydrogels for abdominal tissue regeneration. Sci. Rep. 2017, 7, 2699 10.1038/s41598-017-02962-z. 28578386 PMC5457437

[ref17] Luo X.; Berlin D. L.; Betz J.; Payne G. F.; Bentley W. E.; Rubloff G. W. In situ generation of pH gradients in microfluidic devices for biofabrication of freestanding, semi-permeable chitosan membranes. Lab Chip 2010, 10, 59–65. 10.1039/b916548g. 20024051

[ref18] Choudhary R. C.; Kumaraswamy R. V.; Kumari S.; Sharma S. S.; Pal A.; Raliya R.; Biswas P.; Saharan V. Cu-chitosan nanoparticle boost defense responses and plant growth in Maize (Zea mays L.). Sci. Rep. 2017, 7, 9754 10.1038/s41598-017-08571-0. 28851884 PMC5575333

[ref19] Nie J.; Wang Z.; Hu Q. Chitosan hydrogel structure modulated by metal ions. Sci. Rep. 2016, 6, 36005 10.1038/srep36005. 27777398 PMC5078770

[ref20] Rokugawa I.; Tomita N.; Dobashi T.; Yamamoto T. One-dimensional growth of hydrogel by a contact of chitosan solution with high-pH solution. Soft Mater. 2014, 12, 36–41. 10.1080/1539445x.2012.735316.

[ref21] Dobashi T.; Tomita N.; Maki Y.; Chang C. P.; Yamamoto T. An analysis of anisotropic gel forming process of chitosan. Carbohydr. Polym. 2011, 84, 709–712. 10.1016/j.carbpol.2010.07.004.

[ref22] Kim K.; Ryu J. H.; Lee D. Y.; Lee H. Bio-inspired catechol conjugation converts water-insoluble chitosan into a highly water-soluble, adhesive chitosan derivative for hydrogels and LbL assembly. Biomater. Sci. 2013, 1, 783–790. 10.1039/c3bm00004d. 32481831

[ref23] Kurita K.; Yoshino H.; Nishimura S.-I.; Ishii S. Preparation and biodegradability of chitin derivatives having mercapto groups. Carbohydr. Polym. 1993, 20, 239–245. 10.1016/0144-8617(93)90095-l.

[ref24] Alves N. M.; Mano J. F. Chitosan derivatives obtained by chemical modifications for biomedical and environmental applications. Int. J. Biol. Macromol. 2008, 43, 401–414. 10.1016/j.ijbiomac.2008.09.007. 18838086

[ref25] Zeng R.; Fu H.; Zhao Y. Synthesis of novel biomimetic zwitterionic phosphorylcholine-bound chitosan derivative. Macromol. Rapid Commun. 2006, 27, 548–552. 10.1002/marc.200500876.

[ref26] Yan K.; Xu F.; Wang C.; Li Y.; Chen Y.; Li X.; Lu Z.; Wang D. A multifunctional metal-biopolymer coordinated double network hydrogel combined with multi-stimulus responsiveness, self-healing, shape memory and antibacterial properties. Biomater. Sci. 2020, 8, 3193–3201. 10.1039/d0bm00425a. 32373851

[ref27] Corsello S.; Fulgenzi A.; Vietti D.; Ferrero M. E. The usefulness of chelation therapy for the remission of symptoms caused by previous treatment with mercury-containing pharmaceuticals: a case report. Cases J. 2009, 2, 1626 10.1186/1757-1626-2-199. PMC2783151 19946446

[ref28] Origin of color in complex ions, 2021. https://chem.libretexts.org/@go/page/3707 (accessed May 9, 2021).

[ref29] Spectrochemical series, 2021. https://chem.libretexts.org/@go/page/183320 (accessed May 9, 2021).

[ref30] Bégin A.; Van Calsteren M.-R. Antimicrobial films produced from chitosan. Int. J. Biol. Macromol. 1999, 26, 63–67. 10.1016/s0141-8130(99)00064-1. 10520957

[ref31] Richens D. T. Ligand substitution reactions at inorganic centers. Chem. Rev. 2005, 105, 1961–2002. 10.1021/cr030705u. 15941207

[ref32] Timmons A. J.; Symes M. D. Converting between the oxides of nitrogen using metal-ligand coordination complexes. Chem. Soc. Rev. 2015, 44, 6708–6722. 10.1039/c5cs00269a. 26158348

[ref33] Kaim W.; Das A.; Fiedler J.; Záliš S.; Sarkar B. NO and NO_2_ as non-innocent ligands: A comparison. Coord. Chem. Rev. 2020, 404, 213114 10.1016/j.ccr.2019.213114.

[ref34] Baker A. T. The Ligand Field Spectra of Copper(II) Complexes. J. Chem. Educ. 1998, 75, 98–99. 10.1021/ed075p98.

[ref35] Blake A. J.; Hill S. J.; Hubberstey P. Unique cationic, neutral and anionic copper(II) nitrite species in a single compound. Chem. Commun. 1988, 15, 1587–1588. 10.1039/A803762K.

[ref36] Chandra Maji R.; Mishra S.; Bhandari A.; Singh R.; Olmstead M. M.; Patra A. K. A Copper(II) Nitrite That Exhibits Change of Nitrite Binding Mode and Formation of Copper(II) Nitrosyl Prior to Nitric Oxide Evolution. Inorg. Chem. 2018, 57, 1550–1561. 10.1021/acs.inorgchem.7b02897. 29355312

[ref37] Roger I.; Wilson C.; Senn H. M.; Sproules S.; Symes M. D. An investigation into the unusual linkage isomerization and nitrite reduction activity of a novel tris(2-pyridyl) copper complex. R. Soc. Open Sci. 2017, 4, 170593 10.1098/rsos.170593. 28879000 PMC5579116

[ref38] Beebe D. J.; Moore J. S.; Yu Q.; Liu R. H.; Kraft M. L.; Jo B.-H.; Devadoss C. Microfluidic tectonics: A comprehensive construction platform for microfluidic systems. Proc. Natl. Acad. Sci. U.S.A. 2000, 97, 13488–13493. 10.1073/pnas.250273097. 11087831 PMC17602

[ref39] Sugiura Y.; Hirama H.; Torii T. Fabrication of microfluidic valves using a hydrogel molding method. Sci. Rep. 2015, 5, 13375 10.1038/srep13375. 26300303 PMC4547104

[ref40] Satarkar N. S.; Zhang W.; Eitel R. E.; Hilt J. Z. Magnetic hydrogel nanocomposites as remote controlled microfluidic valves. Lab Chip 2009, 9, 1773–1779. 10.1039/b822694f. 19495462

[ref41] Sershen S. R.; Mensing G. A.; Ng M.; Halas N. J.; Beebe D. J.; West J. L. Independent optical control of microfluidic valves formed from optomechanically responsive nanocomposite hydrogels. Adv. Mater. 2005, 17, 1366–1368. 10.1002/adma.200401239. 34412418

[ref42] Lin S.; Wang W.; Ju X.-J.; Xie R.; Chu L.-Y. A simple strategy for in situ fabrication of a smart hydrogel microvalve within microchannels for thermostatic control. Lab Chip 2014, 14, 2626–2634. 10.1039/c4lc00039k. 24810920

[ref43] Wu J.; Lin Y.; Sun J. Anisotropic volume change of poly(n-isopropylacrylamide)-based hydrogels with an aligned dual-network microstructure. J. Mater. Chem. 2012, 22, 17449–17451. 10.1039/c2jm34010k.

[ref44] Xia Y.; Whitesides G. M. Soft lithography. Angew. Chem., Int. Ed. 1998, 37, 550–575. 10.1002/(sici)1521-3773(19980316)37:5<550::aid-anie550>3.0.co;2-g. 29711088

[ref45] Mata A.; Fleischman A. J.; Roy S. Characterization of polydimethylsiloxane (PDMS) properties for biomedical micro/nanosystems. Biomed. Microdevices 2005, 7, 281–293. 10.1007/s10544-005-6070-2. 16404506

[ref46] Wang J.; Chen W.; Sun J.; Liu C.; Yin Q.; Zhang L.; Xianyu Y.; Shi X.; Hu G.; Jiang X. A microfluidic tubing method and its application for controlled synthesis of polymeric nanoparticles. Lab Chip 2014, 14, 1673–1677. 10.1039/c4lc00080c. 24675980

[ref47] Ianovska M. A.; Mulder P. P. M. F. A.; Verpoorte E. Development of small-volume, microfluidic chaotic mixers for future application in two-dimensional liquid chromatography. RSC Adv. 2017, 7, 9090–9099. 10.1039/c6ra28626g.

