# Supplementary material for: Reversible Crosslinking of Polymer/Metal-Ion Complexes for a Microfluidic Switch
Source: ACS Omega. 2021 Dec 14;6(51):35297–306. doi: 10.1021/acsomega.1c04055 (PMC8717383; doi:10.1021/acsomega.1c04055)
Supplement: Supplementary file 1 — ao1c04055_si_001.pdf [file ao1c04055_si_001.pdf]

Supporting Information

Reversible Crosslinking of Polymer/Metal-Ion Complexes for a Microfluidic Switch

Hojun Lee[†], Soon-Bo Kang[†], Hyunjae Yoo[†], Hae-Ryung Lee[†], and Jeong-Yun Sun [,†,‡]*

[†]Department of Materials Science and Engineering, Seoul National University, 1 Gwanak-ro, Gwanak-gu, 151-744 Seoul, Republic of Korea

[‡]Research Institute of Advanced Materials (RIAM), Seoul National University, 1 Gwanak-ro, Gwanak-gu, 151-742 Seoul, Republic of Korea

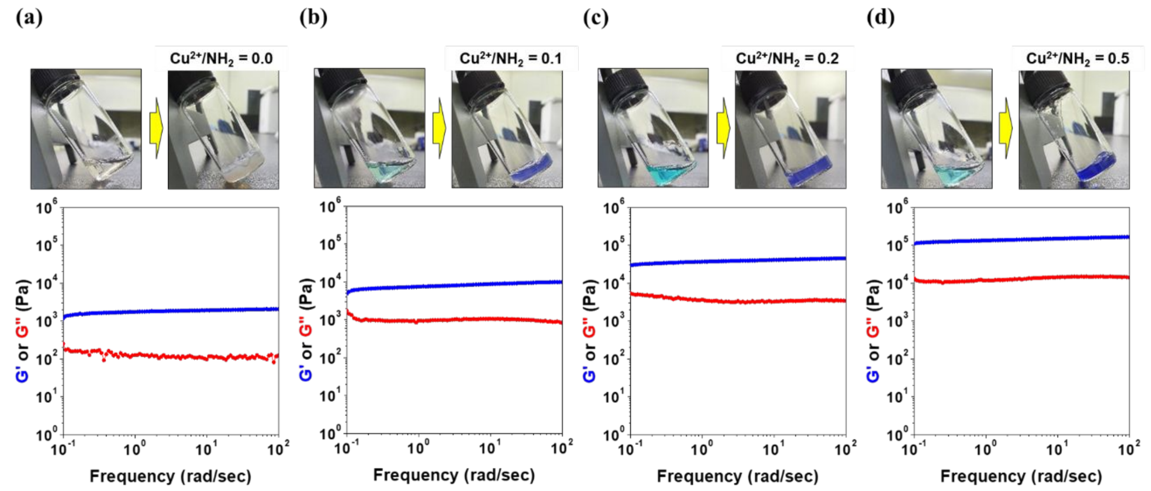

Figure S1. Rheological properties of the chitosan/Cu^{2+} hydrogels with Cu^{2+}/NH_2 (a) 0.0 (b) 0.1 (c) 0.2 (d) 0.5.

Figure S1 shows that the hydrogels were successfully fabricated using a one-dimensional growth system. They did not lose their shape after gelation at pH 12.9 when each vial became atilt (The solution before gelation was prepared at pH 2.7). Also, rheological characterization using dynamic frequency sweep (25℃, 0.5% strain) presented that the values of the storage modulus (G') were much higher than that of the loss modulus (G'') for all the hydrogels. Regarding the effect of Cu^{2+}, the stronger crosslinking was expected through coordination bonding, because the storage modulus of the chitosan/Cu^{2+} hydrogel was higher when the concentration of Cu^{2+} increased.

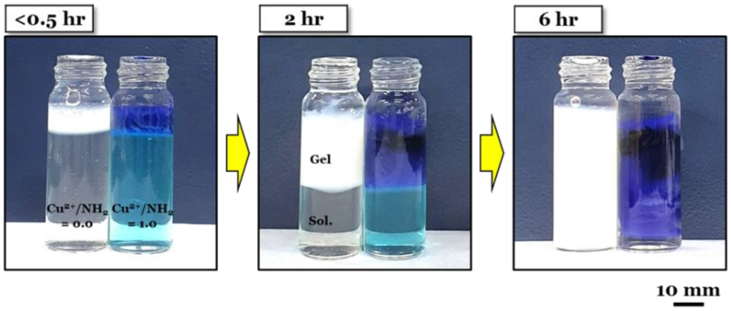

Figure S2. Gelation test of the chitosan/Cu^{2+} hydrogel.

Figure S2 shows a direct comparison of chitosan/Cu^{2+} hydrogel growth outcomes in the 20 wt.% NaOH aqueous solution. The molar ratios (Cu^{2+}/NH_2) between Cu^{2+} and NH_2 were fixed at 0.0 and 1.0, respectively. As a result, the chitosan/Cu^{2+} hydrogel with a large amount of Cu^{2+} (Cu^{2+}/NH_2 = 1.0) grew at the same speed as pure chitosan (Cu^{2+}/NH_2 = 0.0). From this experiment, it was clearly confirmed that the influence of the concentration of Cu^{2+} on the gel formation rate was almost negligible.

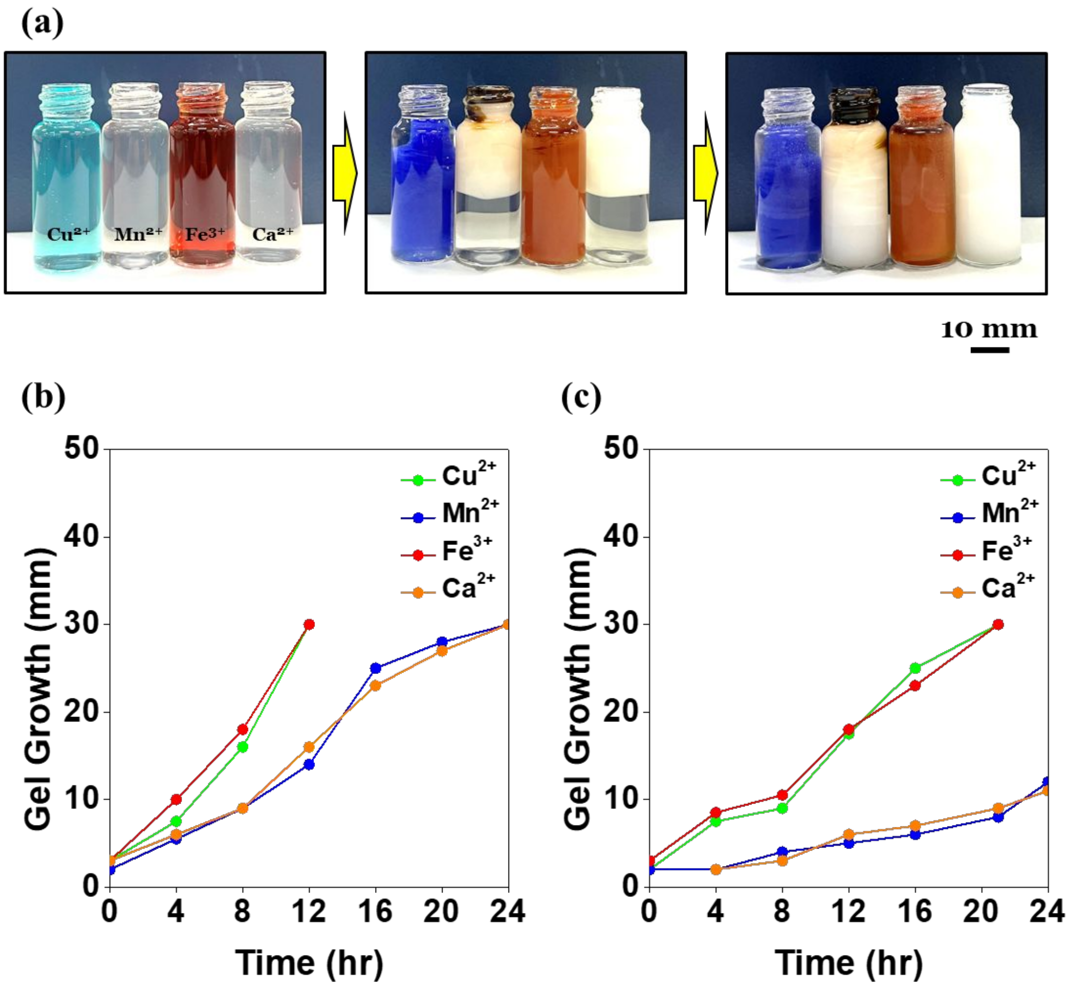

Figure S3. Gelation test of the chitosan/metal-ion (Cu^{2+}, Mn^{2+}, Fe^{3+}, and Ca^{2+}) hydrogel: (a) Photos of growing chitosan/metal-ion hydrogel samples (metal-ion/NH_2 = 0.2) in contact with a 10 wt.% NaOH aqueous solution. (b) Time courses of the chitosan/metal-ion hydrogel (metal-ion/NH_2 = 0.2) with concentrations of 10 wt.% NaOH over a time of 24 hr. (c) Time courses of the chitosan/metal-ion hydrogel (metal-ion/NH_2 = 0.2) with concentrations of 5 wt.% NaOH over a time of 24 hr.

Figure S3 described the chitosan/metal-ion (metal-ion/NH_2 = 0.2, metal-ion: Cu^{2+}, Mn^{2+}, Fe^{3+}, Ca^{2+}) hydrogels fabricated through a one-dimensional gel growth system. The colors of hydrogels were deep blue (Cu^{2+}), creamy white (Mn^{2+}), brown (Fe^{3+}), and white (Ca^{2+}), respectively. Noted that metal chloride compounds ($CuCl_2$, $MnCl_2$, $FeCl_3$, and $CaCl_2$) were used as the source of metal-ions. Then, the gel growth rate in the NaOH aqueous solution (5, 10 wt.%) by metal-ion was evaluated. First, the chitosan/metal-ion hydrogel grew faster as the concentration of NaOH became high. This tendency was valid regardless of metal-ions. Second, the gel growth rate followed the order of $Fe^{3+} \approx Cu^{2+} > Mn^{2+} \approx Ca^{2+}$. This probably resulted in by-product such as $Mn(OH)_2$ and $Ca(OH)_2$, which interrupted the diffusion of OH^-. As explained in the previous work by Nie *et al.*, $Ca(OH)_2$ was observed after chitosan/Ca^{2+} hydrogel formation. The result indicated that the strong affinity of NH_2 to Cu^{2+} encouraged the gel growth.[1]

**Dissolution Time** (min)	**1.0 M Acetate** (pH 3.8~5.6)			**1.0 M Citrate** (pH 3.0~6.2)				
	**pH 4.0**	**pH 5.0**	**pH 5.6**	**pH 3.0**	**pH 4.0**	**pH 5.0**	**pH 5.6**	**pH 6.0**
**Pure Chitosan** (Cu^{2+}/NH_2 = 0.0)	15	60	180	10	N/A	N/A	N/A	N/A
**Chitosan/Cu^{2+}**(Cu^{2+}/NH_2 = 0.2)	60	240	N/A	30	N/A	N/A	N/A	N/A

Table S1. Comparison of aqueous buffer solutions.

Table S1 explains the reason for using acetate buffer solution. Above all, pH range was suitable for a dissolution test of the chitosan hydrogel considering its pKa value of 6.3. Even though citrate buffer solution could also provide the useful pH levels from 3.0 to 6.2, chitosan polymer was not dissolved well probably due to the strong ionic strength of the solution.[2] For example, pure chitosan hydrogel was not perfectly dissolved in citrate buffer solution when pH was above 4.0. Therefore, acetate buffer solution was chosen in order to analyze the effect of pH levels on the dissolution of chitosan/Cu^{2+} hydrogel.

	Chitosan	**Ligand Molecule** (by metal-ion affinity, strong affinity right)			
	**Amine**	**Ethylenediamine**	**2,2′-Bipyridine**	**Nitrite**	**Cyanide**
**Formula**	$\mathbf{N}H_2$	$\mathbf{N}H_2$-CH_2-CH_2-$\mathbf{N}H_2$	$\mathbf{N}C_5H_4$-$C_5H_4\mathbf{N}$	$\mathbf{N}O_2^-$	$\mathbf{C}$≡N^-
**Water Solubility** (25℃, g/ml)	-	1.00	<0.01	0.84 ($NaNO_2$)	>50 (NaCN)
**pKa**	6.3	10.7 (1st)	4.3 (1st)	3.3	9.2

Table S2. Comparison of ligand molecules.

Table S2 describes the method used to select a specific ligand molecule for the sake of enhancing the chitosan/Cu^{2+} hydrogel dissolution process. First, the metal-ion affinity should be stronger than that of NH_2. Briefly, ligand molecules, which bind through relatively electropositive atoms such as N (NO_2^-) and C (CN^-), showed stronger affinity to metal ions than N (NH_2). These ligand molecules tended to show less repulsion between the electrons in the ligand molecules and the electrons in the d-orbitals of the metal ions.[3, 4] As a result, stronger binding with Cu^{2+} could be expected when such molecules were utilized. In general, the ligand molecules were sorted by their metal-ion affinity characteristics, as summarized in **Table S1** (ethylenediamine < 2,2'-bipyridine = nitrite ≪ cyanide).[5] Second, ligand molecules should be soluble in an aqueous solution. Third, ligand molecules should have a low pKa value, as the chitosan polymer is only soluble in an acidic solution.[5-8]

**Dissolution time** (min)	**Chitosan/Cu^{2+}** (Cu^{2+}/NH_2 = 0.2)	**Chitosan/Mn^{2+}** (Mn^{2+}/NH_2 = 0.2)	**Chitosan/Fe^{3+}** (Fe^{3+}/NH_2 = 0.2)	**Chitosan/Ca^{2+}** (Ca^{2+}/NH_2 = 0.2)
**pH 5** (without NO_2^-)	240	300	450	90
**pH 5+NO_2^-** (NO_2^-/metal-ion = 90)	15	25	50	<5

Table S3. Dissolution test of the chitosan/metal-ion (Cu^{2+}, Mn^{2+}, Fe^{3+}, and Ca^{2+}) hydrogel.

Table S3 supports the effect of NO_2^- on the dissolution of the chitosan/metal-ion hydrogels was valid regardless of metal-ions. However, it was notable that metal-ions apparently affected the dissolution rate of the chitosan/metal-ion hydrogels probably due to the different affinity of NH_2 to metal-ion.[9-10] For example, chitosan/Ca^{2+} hydrogel tended to be dissolved faster than others, this was because the weak affinity of NH_2 to Ca^{2+} caused by the absence of the d-orbital (not transition metal).[1]

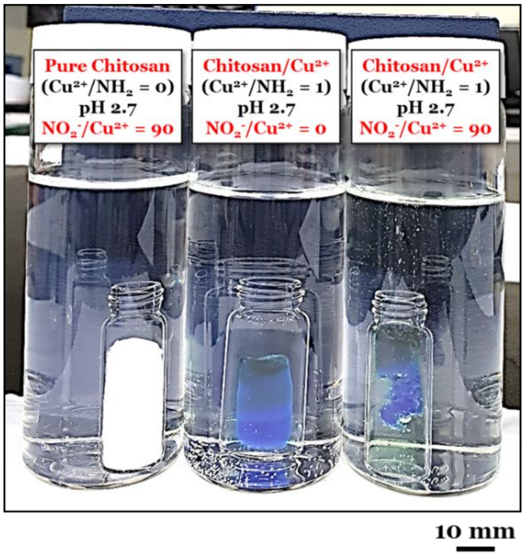

Figure S4. Dissolution test of the chitosan/Cu^{2+} hydrogel.

Figure S4 presents that the use of NO_2^- did not lead to the dissolution of pure chitosan hydrogel ($Cu^{2+}/NH_2 = 0$), while the chitosan/Cu^{2+} hydrogel ($Cu^{2+}/NH_2 = 1$) dissolution process was enhanced when NO_2^- was used. This experiment was implemented in an acetic acid aqueous solution whose initial pH was 2.7, with photos captured 3 min after the dissolution test started. As can be seen, only the chitosan/Cu^{2+} hydrogel immersed in the CH_3COOH/NO_2^- aqueous solution started to dissolve. However, the chitosan/Cu^{2+} hydrogel in the CH_3COOH aqueous solution as well as a pure chitosan hydrogel in the CH_3COOH/NO_2^- aqueous solution remained in the initial state. It should be noted that the cause of the different initial sizes of the hydrogel

between the pure chitosan and chitosan/Cu^{2+} was the shrinkage of the chitosan/Cu^{2+} hydrogel caused by the strong binding during the stabilization process (24 hr).

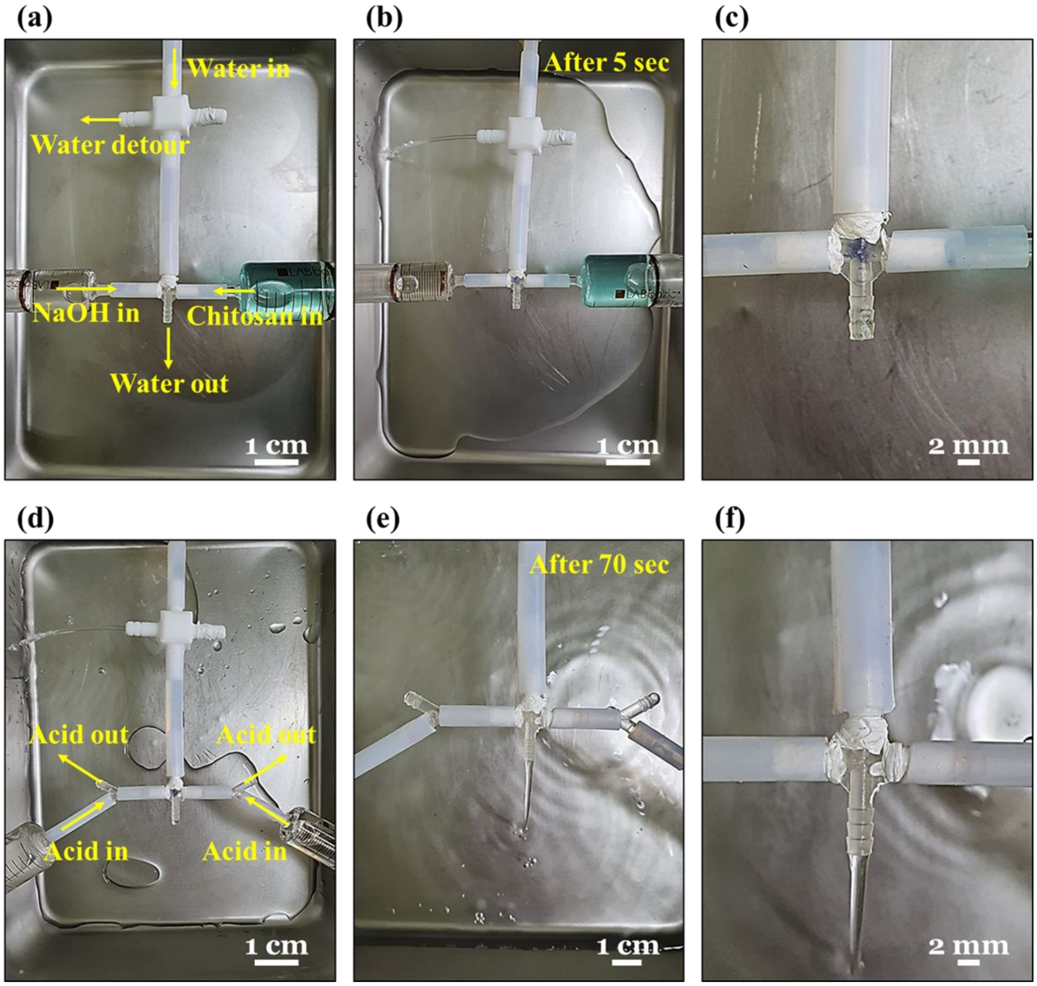

Figure S5. Fluidic switch test using reversible crosslinking: (a) Preparation of the fluidic switch test (b) Close under alkali (c) The blue gel when close (d) Acidic solvents for open (e) Open (f) The blue gel removed when open.

Figure S5 (a) Simple preparation of the fluidic switch test. Three tubes were connected with a four-way connector. A tube was coupled with a water supply (30 ml/min) and the other tubes were connected with syringes. (b) After an injection (20 ml/min) of 0.02 g/ml of the

chitosan/Cu^{2+} solution ($Cu^{2+}/NH_2 = 0.2$) and the 10 wt.% NaOH aqueous solution, the water flow was blocked (5 sec). As indicated, all of the water started to flow along the detour route between the water supply and the four-way connector. (c) The blue gel which formed at the center of the four-way connector was strong enough to endure the solvent pressure. (d) Two different syringes filled with CH_3COOH/NO_2^- aqueous solutions were connected through Y-shaped connectors instead of the chitosan/Cu^{2+} solution coupled with the NaOH aqueous solution. (e) After the injection (20 ml/min) of CH_3COOH (10 wt.%)/NO_2^- (1.5M), the water starts to flow smoothly again (70 sec). (f) The blue gel described in (c) was completely removed.

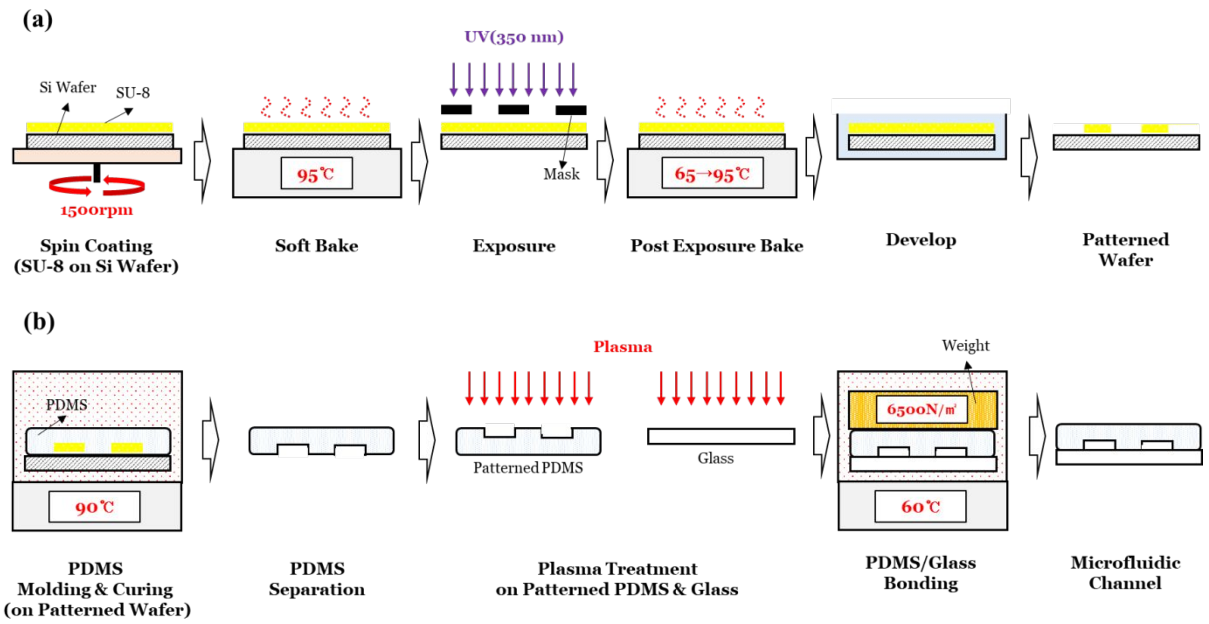

Figure S6. Schematic description of the fabrication of the microfluidic channel: (a) Patterned Si wafer (b) microfluidic channel.

Figure S6 (a) Negative photoresist (SU-8 3025) was spin-coated onto a Si wafer with a thickness of 50 um. After baking for 15 min at 95 °C, 350 nm of UV exposure at a power of 250 mJ/cm^2 was implemented. Then, sequential post exposure baking followed at 65 °C and 95 °C for 1 min and 5 min, respectively. The resultant wafer was immersed in SU-8 developer for 8 min in order to eliminate uncured parts. As a result, a patterned Si wafer was obtained. (b) PDMS was molded onto the patterned Si wafer and cured at 90 °C for 2 hr. After being separated from the Si wafer, the patterned PDMS and glass substrate were attached after a plasma treatment. For strong

bonding between these components, additional heat (60 ℃) and pressure (6500 N/m^2) were applied for 48 hr. Finally, the microfluidic channel was prepared.

REFERENCES

(1) Nie, J.; Wang, Z.; Hu, Q. Chitosan hydrogel structure modulated by metal ions. *Sci. Rep.* **2016**, *6*, DOI: 10.1038/srep36005.

(2) Bégin, A.; Calsteren, M. -R. V. Antimicrobial films produced from chitosan. *Int. J. Biol. Macromol.* **1999**, *26*, 63-67.

(3) Origin of color in complex ions https://chem.libretexts.org/@go/page/3707 (accessed May 9, 2021).

(4) Spectrochemical series https://chem.libretexts.org/@go/page/183320 (accessed May 9, 2021).

(5) Baker, A. T. The ligand field spectra of copper(Ⅱ) complexes. *J. Chem. Educ.* **1998**, *75*, 98-99.

(6) Vrettos, K.; Karouta, N.; Loginos, P.; Donthula, S.; Gournis, D. The role of diamines in the formation of graphene aerogels. *Front. Mater.* **2018**, *5*, DOI: 10.3389/fmats.2018.00020.

(7) Linnell, R. H.; Kaczmarczyk, A. Ultraviolet spectra of [ILL] compounds. *J. Phys. Chem.* **1961**, *65*, 1196-1200.

(8) Wang, Y.; Laborda, E.; Compton, R. G. Electrochemical oxidation of nitrite: Kinetic, mechanistic and analytical study by square wave voltammetry. *J. Electroanal. Chem.* **2012**, *670*, 56-61.

(9) Yan, K.; Xu, F.; Wang, C.; Li, Y.; Chen, Y.; Li, X.; Lu, Z.; Wang, D. A multifunctional metal-biopolymer coordinated double network hydrogel combined with multi-stimulus responsiveness, self-healing, shape memory and antibacterial properties. *Biomater. Sci.* **2020**, *8*, 3193-3201.

(10) Corsello, S.; Fulgenzi, A.; Vietti, D.; Ferrero, M. E. The usefulness of chelation therapy for the remission of symptoms caused by previous treatment with mercury-containing pharmaceuticals; a case report. *Cases J.* **2009**, *2*, DOI: 10.1186/1757-1626-2-199.
